# Cytotoxic Properties of Three Isolated Coumarin-hemiterpene Ether Derivatives from *Artemisia armeniaca* Lam.

**Published:** 2017

**Authors:** Mahdi Mojarrab, Seyed Ahmad Emami, Abbas Delazar, Zahra Tayarani-Najaran

**Affiliations:** a *Pharmaceutical Sciences Research Center, School of Pharmacy, Kermanshah University of Medical Sciences, Kermanshah, Iran. *; b *Department of Pharmacognosy, School of Pharmacy, Mashhad University of Medical Sciences, Mashhad, Iran. *; c *Department of Pharmacognosy, School of Pharmacy, Tabriz University of Medical Sciences, Tabriz, Iran.*; d *Department of Pharmacodynamics and Toxicology, School of Pharmacy, Mashhad University of Medical Sciences, Mashhad, Iran.*

**Keywords:** *Artemisia armeniaca*, Asteraceae leukemic cell lines, apoptosis, coumarin-hemiterpene ether derivatives

## Abstract

Considering multiple reports on cytotoxic activity of the *Artemisia* genus and its phytochemicals, in the current study *A. armeniaca *Lam. and the three components isolated from the plant were subjected to cytotoxic studies. Analytical fractionation of *A. armeniaca *aerial parts for the first time was directed to the isolation of 7-hydroxy-8-(4-hydroxy-3-methylbutoxy) comarin (armenin), 8-hydroxy-7-(4-hydroxy-3-methylbutoxy) comarin (isoarmenin) and deoxylacarol. Cytotoxicity assessed with alamalBlue® assay and apoptosis was detected by PI staining and western blot analysis of Bax and PARP proteins. Extracts and all compounds exhibited cytotoxic activity against apoptosis-proficient HL-60 and apoptosis-resistant K562 cells, with the lowest cytotoxic activity on J774 cell line as non-malignant cell.

Armenin as the most potent component decreased the viability of cell with IC50 of 22.5 and 71.1 µM for K562 and HL-60 cells respectively and selected for further mechanistic study. Armenin increased the sub-G1 peak in flow cytometry histogram of HL-60 and K562 treated cells and increase in the amount of Bax protein and the cleavage of PARP in comparison with the control after treatment for 48 h in K562 treated cells verified the apoptotic activity of the armenin.

Taken together, according to the finding of this study armenin was introduced as a novel cytotoxic compound with apoptotic activity, which is encouraging for further mechanistic and clinical studies.

## Introduction

Cancer chemotherapy is mainly owed to the compounds of natural origin. Naturally derived anticancer compounds have been applied directly or as new leads in the discovery of novel structures with anticancer action. Newer generation of cancer chemotherapeutics are selected according to their efficacy and less toxicity profiles. Demanding high potential compounds with the lowest toxicity is the base for screening compounds with the highest potential in clinical implications (1). 

Afterward elucidation of the molecular signaling events elicited by the compounds would give insight to the mechanistic pathways evoked by phytochemicals. Understanding the fact that most anticancer chemicals interfere with programmed cell death propelled most studies to clarify the complex cascade of events that have occurred in apoptosis. In this regards many plants and phytochemicals are proved to be inducers of apoptosis as cancer preventive or chemotherapeutics (2).

*Artemisia armeniaca* Lam., locally known as Dermane ye Armanestani, is an evergreen or semi-evergreen sub-shrub of the genus *Artemisia* L. (Asteraceae), which grows abundantly in the Arasbaran area of Eastern Azarbaijan province, northwest of Iran (3). Former phytochemical studies on the aerial parts of *A. armeniaca *resulted in the identification of simple and prenylated coumarins (4-7) as well as some flavonoids (7). α-Pinene and 1,8-cineole have been reported to be the major constituents of the obtained hydro-distilled oil from the aerial parts of this plant (8) while non-terpene hydrocarbons were identified as the main components of the essential oil in another study (9). Coumarins include a very large class of secondary metabolites observed throughout the plant kingdom (10). Biological effects of coumarin and related derivatives, such as anti-thrombotic and vasodilatory (11), anti-bacterial (12), anti-mutagenic (13) and cancer chemoprevention as well as anti-tumour (14-18), seem to originate from the coumarin nucleus (16, 19). 

The current study aimed to evaluate the *in-vitro* cytotoxic and apoptogenic potential of MeOH and CH_2_Cl_2_ extracts besides three previously reported coumarins from* A. armeniaca* on two human apoptosis-proficient HL-60 and apoptosis-resistant K562 cancer cells. Extraction and isolation of the compounds were accomplished using the previously described method for purification of coumarin-hemiterpene ether derivatives (7). 

## Experimental

*Reagents and chemicals*

AlamarBlue^®^ (resazurin) from Sigma (Saint Louis, MO, USA); (RPMI-1640) and FCS from Gibco; Bax (Cat number: 2772), β-actin (Cat number: 4970) and PARP (Cat number: 9542) antibodies, anti-rabbit IgG and HRP linked antibody from CellSignaling technology (Boston, USA); ECL Western blotting detection reagent from Bio-RaD (USA); the fluorescent probe propidium iodide (PI), protease inhibitor cocktail, phosphatase inhibitor cocktail, sodium citrate, Triton X-100, phenylmethylsulfonyl fluoride and Bio-Rad Protein Assay Kit (Hercules, CA); all the solvents used in the extraction and purification procedures were of gradient grade and purchased from Scharlau (Spain) and Caledon (Canada).

*Plant materials*

The aerial parts of *A. armeniaca* Lam. were collected from Arasbaran, East Azarbaijan province (Iran) during August 2008. The plant was identified by Mr. AH Talebpour from Tabriz University of Medical Sciences. Voucher specimen (No. TBZfph 528) was deposited in the Herbarium of School of Pharmacy, Tabriz University of Medical Science, Tabriz, Iran.

*Instrumentation*

Vacuum Liquid Chromatography was performed on Sep-Pak Vac 35 cc (10 g) C18 cartridges (Waters). Reversed- phase semi-preparative HPLC separations were carried out on a Wellchrom Knauer system (Herbert Knauer GmbH, Berlin, Germany), consisting of a Knauer K-1800 pump, Dr. Maisch GmbH ODS column (250 mm × 16 mm, 10 µM) and a Knauer K-2800 UV-Vis detector. NMR spectra were recorded on a Bruker Avance 400 MHz in CD_3_OD as the solvent and residual solvent peaks used as internal standard. ESIMS data were obtained on a Finnigan MAT95 spectrometer.

*Extraction and purification*

The dried and ground aerial parts of *A. armeniaca *(100 g) were extracted in a Soxhlet apparatus with CH_2_Cl_2 _and MeOH (1.5 L each) for 20 and 24 h, successively.

The extracts were separately dried under reduced pressure at a maximum temperature of 45 ºC to yield 10.9 and 7.1 g of CH_2_Cl_2_ and MeOH extracts, respectively.

A portion of the methanol extract (2×2 g) was subjected to a Vacuum liquid chromatography (VLC) system with H_2_O containing increasing amounts of MeOH (10%, 20%, 40%, 60%, 80% and 100%) to give six fractions (A, B, C, D, E & F) respectively.

**Figure 1:**
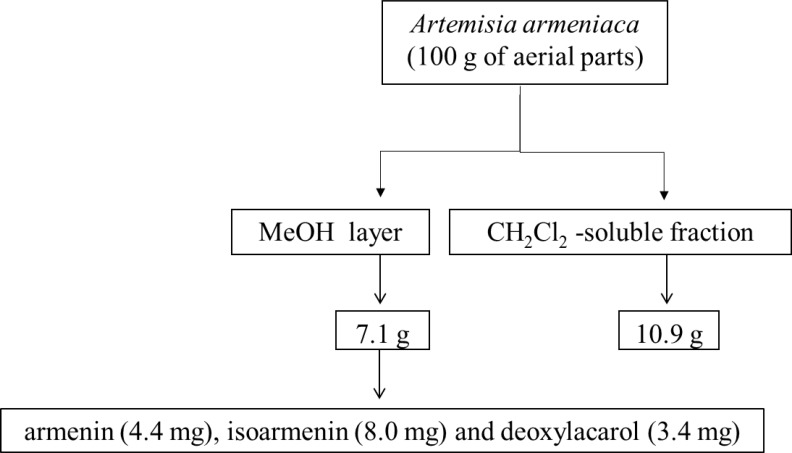
Partitioning scheme of *A. armeniaca*

Further purification of the fraction C by semipreparative HPLC (mobile phase: 0-60 min, MeOH from 40 to 60% in H_2_O; 60-64 min, 60% MeOH in H_2_O; 64-68 min MeOH from 60 to 100% in H_2_O; 68-75 min 100% MeOH, flow rate 8 mL/min, detection at 220 nm) yielded three prenylated coumarins including armenin (4.4 mg; t_R _= 43.8 min), isoarmenin (8.0 mg, t_R_ = 46.2 min) and deoxylacarol (3.4 mg, t_R_ = 55.8 min). Structure elucidation of armenin (7-hydroxy-8-(4-hydroxy-3-methylbutoxy)-2H-chromen-2-one), isoarmenin (8-hydroxy-7-(4-hydroxy-3-methylbutoxy)-2H-chromen-2-one)and deoxylacarol (8-(4-Hydroxy-3-methylbutoxy)-7-methoxy-2H-chromen-2-one) was achieved using spectroscopic techniques including ESIMS and proton NMR experiments (Figure 1 and Table 1). Retention time values as well as the obtained spectroscopic data (as follows) were in good agreement with those given in the literature (5, 7).

Armenin (1). White amorphous powder. ^1^H-NMR (CD_3_OD, 400 MHz): 0.92 (3H, d, *J *= 6.5 Hz, Me-5’), 1.52 (1H, m, H-2´a), 1.82 (1H, m, H-2’b), 1.88 (1H, m, H-3´), 3.36 (1H, m, H-4´a), 3.45 (1H, m, H-4´b), 4.18 (2H, m, H-1´), 6.12 (1H, d, *J *= 9.5 Hz, H-3), 6.78 (1H, d, *J *= 8.8 Hz, H-6), 7.14 (1H, d, *J *= 8.8 Hz, H-5), 7.77 (1H, d, *J *= 9.5 Hz, H-4).

ESIMS: m/z 265.1 [M+H]^+^, 287.2 [M+Na]^+^.

Isoarmenin (2). White amorphous powder. ^1^H-NMR (CD_3_OD, 400 MHz): 1.00 (3H, d, *J *= 6.5 Hz, Me-5’), 1.56 (1H, m, H-2´a), 1.96 (1H, m, H-2’b), 2.07 (1H, m, H-3´), 3.36 (1H, m, H-4´a), 3.49 (1H, m, H-4´b), 4.19 (2H, m, H-1´), 6.26 (1H, d, *J *= 9.5 Hz, H-3), 7.02 (1H, d, *J *= 8.8 Hz, H-6), 7.09 (1H, d, *J *= 8.8 Hz, H-5), 7.90 (1H, d, *J *= 9.5 Hz, H-4).

ESIMS: *m/z *265.1 [M+H]^+^, 287.2 [M+Na]^+^.

Deoxylacarol (3). White amorphous powder. ^1^H-NMR (CD_3_OD, 400 MHz): 1.08 (3H, d, *J *= 6.7 Hz, Me-5’), 1.52 (1H, m, H-2´a), 1.82 (1H, m, H-2’b), 2.03 (1H, m, H-3´), 3.52 (1H, m, H-4´a), 3.56 (1H, m, H-4´b), 3.99 (3H, s, methoxy), 4.17 (2H, m, H-1´), 6.31 (1H, d, *J *= 9.5 Hz, H-3), 7.10 (1H, d, *J *= 8.7 Hz, H-6), 7.41 (1H, d, *J *= 8.7 Hz, H-5), 7.93 (1H, d, *J *= 9.5 Hz, H-4).

ESIMS: *m/z *279.2 [M+H]^+^, 301.2 [M+Na]^+^.

*Cell cultures and treatment agents*

The human leukemic cancer cell lines HL-60 and K562 were obtained from Pasteur Institute (Tehran, Iran) and maintained in RPMI-1640 medium with 10% v/v fetal bovine serum and 100 u/mL penicillin and 100 mg/mL streptomycin at 37 °C in a humidified atmosphere of 5% CO_2_ and 95% of air.

*In-vitro cell viability*

AlamarBlue^®^ is an indicator dye which incorporates an oxidation-reduction (REDOX) indicator that both fluoresces and changes colour in response to the chemical reduction of growth medium, resulting from cell growth. The alamarBlue^®^ assay is designed to quantitatively measure the proliferation of various human and animal cell lines, bacteria and fungi. Upon entering cells, the blue and non florescent resazurin convert to the florescent and purple resorufin in viable cells (20). About 5×10^4 ^K562 and 10^5 ^HL-60 cells were seeded in each well of 96-microwell plate and treated with various concentrations of each extracts (0-200 µg/mL) and armenin, isoarmenin and deoxylacarol (0-200 µM). J774 cell line was used as non-malignant cells. After 48 incubation, 20 µL resazurin (0.01% w/v in PBS) was added to each well and the plates were incubated at 37 ºC for 4 h before the absorbance was measured at 570 nm (test wavelength) and 600 nm (reference wavelength) in a Synergy H4 Hybrid Multi-Mode Microplate Reader (BioTek, Winooski, USA). The cytotoxicity of the pure compounds was expressed as IC_50_, calculated using Prism 5 Software (GraphPad, La Jolla, CA, USA) and presented as mean ± SD from three independent experiments (with three replicates for each concentration tested extract). For each study, a control sample remained untreated and received only medium in place of the text materials. Paclitaxel 1 µM was used as positive control. 

*PI staining*

Apoptotic cells were detected by PI staining of small fragments of DNA in treated cells followed by flow cytometry. It has been reported that following DNA fragmentation the so-called sub-G1 peak can be noticed following incubation of cells in a hypotonic phosphate-citrate buffer containing quantitative DNA-binding dye such as PI. Apoptotic cells that have lost DNA will take up less stain and will show up in the left side of the G1 peak in the histogram. Briefly, 10^6 ^K562 and HL-60 cells were seeded in each well of a 24-well plate and treated with armenin in different concentrations (0, 25 and 50 µM) for 48 h. Floating and adherent cells were then harvested and incubated at 4 °C overnight in the dark with 750 μL of a hypotonic buffer (50 μg/mL PI in 0.1% sodium citrate plus 0.1% Triton X-100) before flow cytometric analysis using a FACScan flow cytometer (Becton Dickinson, San Diego, CA) was performed. A minimum of 10^4^ events was acquired for each sample. All data were then analyzed using WinMDI version 2.8 software.

*Western blot analysis*

About 10^7 ^HL-60 and K562 cells were treated with 12.5, 25 and 50 μM of armenin, isoarmenin and deoxylacarol (0-200 µM) for 48 h. The cells rinsed and harvested with cool PBS for 3 times, the cell pellet was resuspended in a lysis buffer containing 50 mM Tris-HCl (PH 7.4), 150 mM NaCl, 1% TritonX-100, 1 mM EDTA, 0.2% SDS, 1% Protease inhibitor cocktail, 1% phosphatase inhibitor cocktailand 1 mM phenylmethylsulfonyl fluorideand left on ice for 30 min. After centrifugation at 10000 rpm for 20 min at 4 °C, the cell lysate was collected and protein concentration was determined according to the Bio-Rad Protein Assay kit. Equal amount of proteins were subjected to 12% SDS-page (W/V). The proteins were transferred to a polyvinylidene fluoride (PVDF) membrane and subjected to immunoblotting using Bax, β-actin and, PARP antibody as primary antibodies and anti-rabbitIgG and HRP-linked antibody as secondary antibodies, Bax protein band and PARP cleavage in K562 and HL-60 cells were detected by enhanced chemiluminescenceusing the ECL western blotting detectin reagent. Images were quantified using Gel-pro Analyser V.6.0 Gel Analysis software (Media Cybernetics, InC, Bethesda, MD).

*Statistical analysis*

One way analysis of variance (ANOVA) and Bonferroni posthoc test were used for data analysis. All the results were expressed as mean±SD and *p* values below 0.05 were considered statistically significant.

## Results

*Cytotoxicity of isolated compounds*

**Figure 2:**
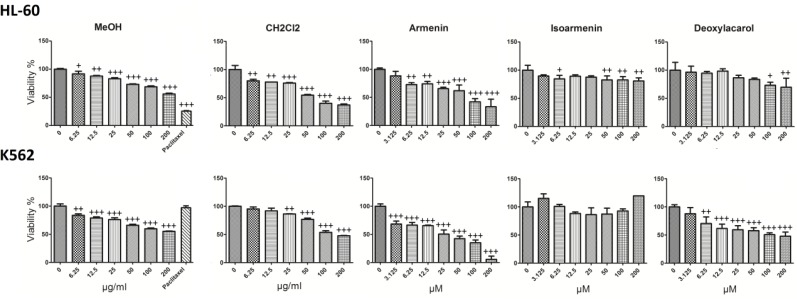
The concentration-dependent effects of CH2Cl2 and MeOH extracts (0-200 μg/ml) and 7-hydroxy-8-(4-hydroxy-3-methylbutoxy) comarin (armenin), 8-hydroxy-7-(4-hydroxy-3-methylbutoxy) comarin (isoarmenin) and deoxylacarol on the viability of K562 and HL-60 cells. Among three compounds armenin exhibited high cytotoxic activity against apoptosis-proficient HL-60 and apoptosis-resistant K562 cells, with IC50 values ranging from 22.5 to 71.1 μM with much less cytotoxic effects on normal human lymphocytes. Paclitaxel 1 μM was used as positive control. Values were mean±SD of at least three independent experiments, each in triplicates. +*P *< 0.05, ++*P *< 0.01 and +++*P *< 0.001 compared to control.

Armenin, isoarmenin and deoxylacarol were examined for cytotoxic potential on K562, HL-60 and normal cells (J774). Cells were incubated at 37 °C and 5% CO_2_ with various concentrations of the CH_2_Cl_2 _and MeOH extracts (0-200 µg/mL) and compounds (0-200 µM) for 48 h. Results demonstrated that extracts decreased cell viability in a concentration-dependant manner (Figure 2). Among all the samples, armenin demonstrated the most cytotoxic effects on cancer cells, with limited adverse effect on normal cells. IC_50_ values (µM) for different extracts of armenin in HL-60 and K562 cells are presented in Table 2. Paclitaxel 1 µM was used as positive control and decreased the viability of K562, HL-60 to 25.5 ± 0.5 and 96.3 ± 1.5 respectively at this concentration.

**Figure 3:**
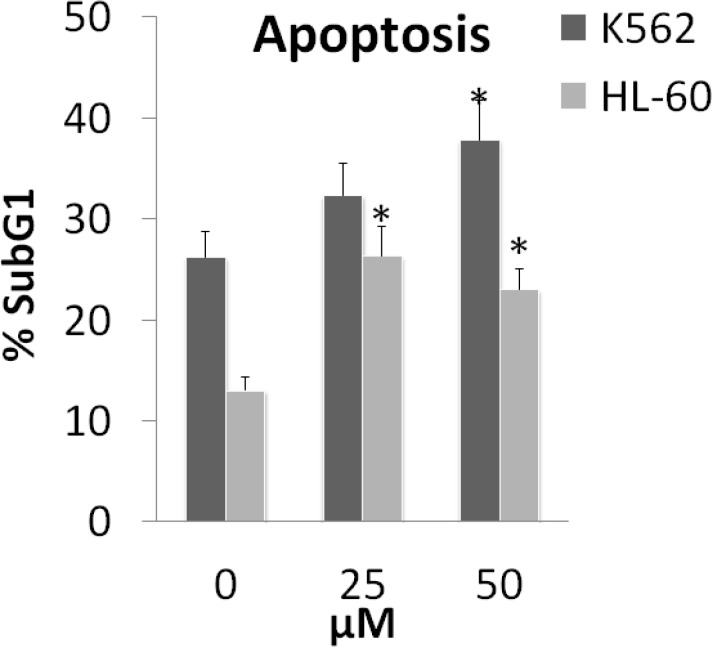
Percentage of sub-G1 peak after PI staining and flow cytometry analysis of armenin (0.0, 25 and 50 µM) showing apoptosis induction in K562 and HL-60 cells. All experiments were done in triplicate. *P < 0.05 compared to control

**Figure 4:**
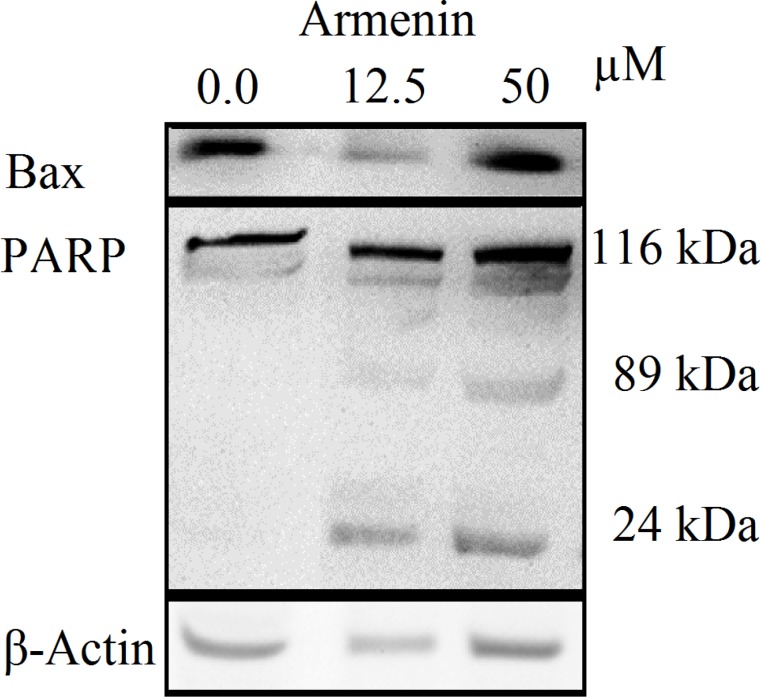
Proteolytic cleavage of poly (ADP-ribose) polymerase (PARP) in K562 cells after 48 h exposure to armenin (0.0, 12.5 and 50 µM). β-Actin was used as a loading

**Table 1:** Chemical structure of armenin, isoarmenin and deoxylacarol

**Compound name**	**R1**	**R2**
Armenin	OH	4-Hydroxy-3-methylbutoxy
Isoarmenin	4-Hydroxy-3-methylbutoxy	OH
Deoxylacarol	OCH_3_	4-Hydroxy-3-methylbutoxy

**Table 2:** IC_50_ values for CH_2_Cl_2 _and MeOH extracts (µg/mL) and 7-hydroxy-8-(4-hydroxy-3-methylbutoxy) comarin (armenin), 8-hydroxy-7-(4-hydroxy-3-methylbutoxy) comarin (isoarmenin) and deoxylacarol (µM) in HL-60 and K562 cell lines

	**extracts**		**Pure compounds**		
**Cell Line**	**CH**_2_ **Cl**_2_	**MeOH**	**armenin**	**isoarmenin**	**deoxylacarol**
K562	130.7	155.4	22.5	>200	108.9
HL-60	74.95	>200	71.1	>200	>200

*Apoptosis induction by isolated compounds*

Apoptosis in K562 and HL-60 cell lines was detected with flow cytometry using PI staining test. Cells incubated with various concentrations (0, 25 and 50 µM) of armenin for 48 h*.* Sub-G1 peak of treated cells in flow cytometry histograms compared to that (=Sub-G1 peak) of untreated control cells revealed the induction of apoptosis in treated cells (Figure 3).

*Effect of armenin on poly (ADP ribose) polymerase cleavage*

Since K562 cells were more sensitive to armenin K562 cells were selected for western blot analysis. The increase in the amount of Bax and cleavage of PARP-1 116 kDa to 89 and 24 kDa fragments was used as an indicator of apoptosis. In K562 cells, the amount of Bax was increased and PARP-1 was cleaved clearly to the 89 kDa and 24 kDa fragments after treatment with armenin (0.0, 12.5 and 50 µM) after 48 h (Figure 4). 

## Discussion

We have previously demonstrated the potent cytotoxic and apoptotic activity of the CH_2_Cl_2_ extract of *A. armeniaca* in K562 and HL-60 cells (21). In this regard, we have chosen the plant for further analytical and biological assays. In this study, we have examined the cytotoxic properties of CH_2_Cl_2 _and MeOH extracts (0-200 µg/mL) and armenin, isoarmenin and deoxylacarol isolated from *A. armeniaca. *The results show that the reduced viability of cancer cells treated with armenin is accompanied by apoptosis induction verified by sub G1 peak in the flowcytometery histogram and PARP cleavage of treated cells. We therefore infer that among three pure compounds, armenin is a potent cytotoxic and apotogenic one that could triggers at least certain aspects of the apoptosis signaling pathway, which activates apoptotic mediators that are closely associated with the levels of DNA damage and degradation of active DNA repairing enzymes, PARP (22). PARP cleavage can be seen by not only in K562 cells with IC_50_ values of armenin, but also in lower concentrations of the compound. Although K562 cells are apoptosis resistant (23), our findings suggest this cell line is more sensitive to armenin than HL-60 cells. This controversy may result from different pathways affected by armenin in K562 and HL-60 cells. Armenin’s cytotoxic mechanism on cancer cells is still not fully undrestood. The difference in the cytotoxic activity of armenin, isoarmenin and deoxylacarol may be linked to other factors such as side chains, chiral structure, lipophilicity, and molecular or electrical features, affecting the affinity of the molecule to the active site of action.

Cytotoxic and apoptogenic properties of different extracts of various species from the genus *Artemisia *have been studied so far (24-27) and have resulted in the isolation of some known coumarins like scopoletin and isoscopoletin from the active extract (25). A previous study (28) has revealed the cytotoxic activity of isoscopoletin against lung and colon cancer cell lines. Cytotoxic and cytostatic effects of scopoletin on tumoural lymphocytes had already been reported as well (29).

There are reports that prenylated coumarins can exhibit cytotoxic activity. Ferulenol, a prenylated 4-hydroxycoumarin from *Ferula communis* with remarkable dose-dependent cytotoxicity against various human tumor cell lines, displayed stimulation of tubulin polymerization *in-vitro* and rearrangement of cellular microtubule network as well as alteration in nuclear morphology (30). Based on another study, ferulenol possessed cytotoxic properties on hepatocytes. Additionally, the prenylated coumarin reduced factor X biosynthesis at non-cytotoxic concentrations. 

The prenyl residue played a major role in ferulenol activity (31). A study on the anti-leukemic activities of a series of prenylated coumarins isolated from *Toddalia asiatica *(L.) Lam. indicated that the most potent cytotoxic and anti-proliferative compound displayed a dual effect as a cell differentiating agent and apoptosis inducer in U-937 cells (32). Minutin B, a monoterpene coumarin from *Micromelum minutum* Wight & Arn. leaves exhibited strong cytotoxic activity against four lung adenocarcinoma cell lines including A549, SBC3, K562, and K562/ADM (33). 

## Conclusion

We have previously demonstrated that CH_2_Cl_2_ extract of *A. armeniaca* is a highly potent cytotoxic agent against K562 and HL-60 cells with minimal effect on normal cells. Herein we have done additional cytotoxic and apoptogenic assays on three pure compounds isolated from *A. armeniaca *on human leukemia cell lines and human normal cells. Based on this study the cytotoxic and apoptogenic activity of armenin was confirmed. It is also worthy to emphasize that the high antileukemic activity of armenin was accomplished with low toxicity to human normal cells. Thus, it is imperative to continue the pursuit of new therapeutic aspects of armenin as a novel anticancer agent and it may be beneficial to discover multiple signaling pathways affected by the compound for clinical usage.

## Conflict of interest

The authors declare that there are no conflicts of interest.
